# An Evaluation of Global Hazard Communication with Ethical Considerations

**DOI:** 10.1371/currents.dis.47581b109e865f7b64d831f86a7fd7f4

**Published:** 2018-08-07

**Authors:** Thomas Richardson, Gemma Hayward, Kevin Blanchard, Virginia Murray

**Affiliations:** Intern, Public Health England, London, United Kingdom; Intern, Public Health England, London, United Kingdom; Global Disaster Risk Reduction, Public Health England, London, United Kingdom; Public Health England, London, England; UNISDR Scientific and Technical Advisory Group, Geneva, Switzerland; Integrated Research on Disaster Risk Scientific Committee, Beijing, China

## Abstract

Introduction: Despite the large number of hazards occurring every year, it is often only the most catastrophic and rapidly occurring hazards that are covered in detail by major news outlets. This can result in an under-reporting of smaller or slowly evolving hazards such as drought. Furthermore, the type or country in which the hazard occurs may have a bearing on whether it receives media coverage. The Public Health England (PHE) global weekly hazards bulletin is designed to inform subscribers of hazards occurring in the world in a given week regardless of location or type of natural hazard. This paper will aim to examine whether the bulletin is reporting these events in a way that matches a number of international disaster databases.  It will also seek to answer if biases within media outlets reporting of an event is impacting on the types of hazards and events being covered.  Through the analysis of data collected, it is hoped to be able to consider the ethical implications of such a bulletin service and provide recommendations on how the service might be improved in the future.

Methods: The study used a year’s worth of global hazards bulletins sent by Public Health England.  These bulletins aim to communicate hazards in the form of compiled articles from news outlets around the world. Data from these bulletins was collected and analysed by hazard type and the country in which hazards occurred.  It was then compared to recognised hazard databases to assess similarities and differences in the hazards being reported via media or through dedicated hazard databases. The recognised hazard databases were those run by the Emergency Events Database (EM-DAT), European Civil Protection and Humanitarian Aid Operations (ECHO) and National Aeronautics and Space Administration (NASA) respectively.

Results: The PHE bulletin overall was found to be comparable to other global hazard or disaster databases in terms of hazards included by both country and type of hazard. The PHE bulletin covered a greater number of unique hazard events than the other databases and also covered more types of hazard. It also gave more frequent coverage to the United Kingdom and Canada than the other databases, with other countries appearing less frequently. More generally, the PHE bulletin and the databases it was compared to appear to focus more on hazards either occurring in developed countries or fast-onset ones such as landslides or floods. On the other hand, slow-onset hazards such as drought or those occurring in developing countries appear to be under-reported and are given less importance in both the bulletin and databases.

Discussion and recommendations: We recommend that the resources compared review their inclusion criteria and assess whether the discrepancies in hazard type and country can be ratified through changes in how hazards are assessed for inclusion. More research should be undertaken to assess whether similar findings arise when comparing databases in other areas within the remit of public health.

## Introduction

**Background**

There have been over 6000 natural and technological hazards over the past 10 years resulting in over 700 000 deaths 1. Indeed, in 2015 alone, there were 376 natural hazards and over 22 000 lives lost as a result 2. Moreover, as well as the human lives lost, the economic impact of these disasters can be enormous with the total economic losses of an event such as Hurricane Katrina reaching in excess of $125 billion 3.

Given the large number of hazards occurring every year, it is often only the most catastrophic and rapidly occurring ones that are covered in detail by major news outlets 4^,^ 5. This can result in an under-reporting of smaller or slow-onset hazards such as drought 6. There is evidence to suggest that some hazards are prioritised over others and that the hazard type or country in which the hazard occurs can have a bearing on this 7^,^ 8^,^ 9^,^ 10. Moreover, critics argue that there are numerous crises neglected by the mainstream media and that there is a bias towards hazards occurring in developed countries 11^,^ 12^,^ 13^,^ 14. For the purposes of this study we used the UN country classification system in order to provide a standardised definition of 'developed' 15.

Whilst it may be unfair and even impossible to force news outlets to report on all hazards, the under-reporting in the media can then have an impact on the response to the hazard itself. This may be through less international humanitarian aid being given due to less awareness of the hazard or a worse national response as the lack of press coverage may make a government less accountable for their hazard response 16^,^ 17.

This poses an ethical conundrum when disseminating information regarding these hazards. If the global weekly hazards bulletin was found to favour certain hazards or countries over those recorded on international disaster databases, one could argue that the aim of the bulletin - to inform - has not been met fully. This article therefore provides an opportunity to examine the resource in more detail and suggest areas which could be improved. Moreover, on a larger scale, these improvements may be applicable to similar communication tools produced by other agencies worldwide whether they cover hazards or not 18^,^ 19^,^ 20.

The global hazards weekly bulletin at Public Health England (PHE) was designed to provide an easy to use, informative weekly guide on natural and technological hazards that have occurred in the previous week 21. Information is gathered via a Google Alerts system with over thirty search terms used to identify any news articles on any hazard occurring globally (see Appendix 1). These articles are then sorted into individual hazards and each is reviewed for any political or other reporting bias. This review process is subject to a specified criteria and guidance (see Appendix 2). For example, any articles that are not from a reputable source, or have an overtly political stance, are removed. The resulting information is then sorted into a list of hazards broken down by country and provides the article’s headline with a hyperlink embedded which allows the reader to click and view the source directly. There are currently over 6000 subscribers to the service from around the world.

**Objectives**

-The objectives of our research are as follows:

-To establish whether the bulletin achieves its aims of informing the public of hazards occurring throughout the world;

-To identify any reporting bias such as under or over-reporting of specific hazards in particular countries through a comparison with three global and internationally recognised disaster databases run by EM-DAT, European Civil Protection and Humanitarian Aid Operations (ECHO) and National Aeronautics and Space Administration (NASA) respectively 22^,^ 23^,^ 24;

- To highlight the possible ethical consequences of any bias and assess ways of reducing this to improve the bulletin such as altering the alerts system or news sources used.

## Methods

The authors analysed all PHE global hazard bulletins produced over a twelve-month period from the 1st July 2016 to the 31st June 2017. Data was extracted on the following areas:

- PHE Bulletin date
- Principle country in which the hazard took place
- Type of hazard (as defined in the EM-DAT hazard classification) 22
- Article source
- Article source country

Duplicate articles (those concerning the same hazard event in a specific country) were excluded from the data analysis. We noted the number of articles on each specific hazard type. We then compared this data with the Centre for Research on the Epidemiology of Disasters (CRED) Annual Disaster Statistical Review 2015 and to the three recognised hazard databases - EM-DAT, ECHO and NASA, as well as the International Federation of Red Cross (IFRC) World Disasters Report, which allowed us to identify whether the PHE bulletin is picking up smaller hazards that may not be included in the global databases 1^,^ 2^,^ 22^,^ 23^,^ 24

The data compared was limited to the date of the hazard, the principle country in which the hazard took place and the hazard type.

It is important to note that it was decided to remove technological hazards and industrial accidents from our comparison. The bulletin editor had stated during the collection of data and its analysis that the clear majority of technological hazards were not published in the bulletin due to the need to maintain political neutrality and meet the Civil Service Code 25. These hazards tend to be more politicised; industrial accidents, for example, tend to have a high degree of blame attached to them within media articles 13.

## Results

Our dataset included 1012 individual articles on 579 events which had been published in the PHE bulletin from 1st July 2016 to 30th June 2017. Data from the same timeframe was extracted on 227 hazards on the EM-DAT database, 461 hazards on the ECHO database and 124 from NASA. The PHE bulletin included 54%, 53% and 63% of all hazards contained within EM-DAT, ECHO and NASA databases respectively. 39.3% of hazards from the other three databases were not included in the PHE bulletin.

**Comparison of type of hazard**

Data was extracted on the type of hazards in each database. All types of hazard were classified according to the EM-DAT classification of natural disasters to ensure comparability between databases. Table 1 illustrates the range and number of hazards within each of the four databases. The PHE bulletin and ECHO databases both had the largest range of types of hazards with 20 and 17 individual hazard types respectively. Flooding was by far the most commonly occurring hazard covering 21.2% of PHE Bulletin, 39.2% of EM-DAT and 28.2% of ECHO hazards. In comparison, wildfires were the most common hazard on the NASA database accounting for 23.4% of all hazards with flooding the sixth most prevalent (9.7%).

**Table 1:**
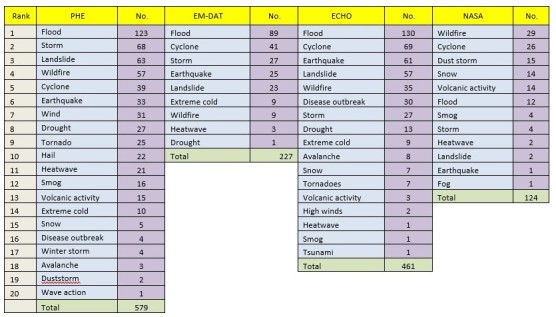
Number of unique hazards ranked by type of hazard in the PHE bulletin, EM-DAT, ECHO and NASA databases respectively.

The PHE Bulletin alone was assessed for the number of articles included per individual disaster type as these results are depicted in Table 2. Across all 579 unique hazards, there were a total of 1012 articles included in the bulletin giving a mean of 1.75 articles per individual hazard. Focusing on the most common hazards, there was a mean of 2.94 articles on each cyclone covered in the bulletin compared to 2.01, 1.68 and 1.24 for each flood, landslide and storm respectively.

**Table 2:**
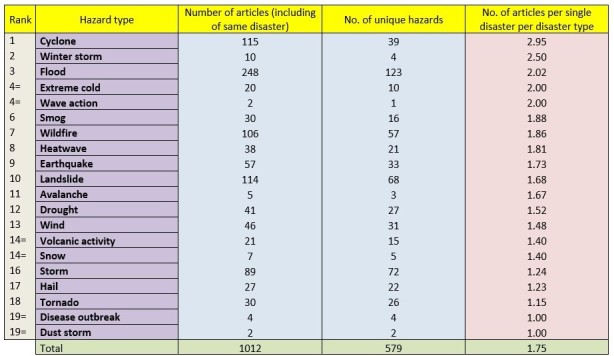
Ranking of the number of articles per unique hazard per disaster type within the PHE bulletin.

**Comparison of country of hazard**

Data was also extracted on the countries in which these hazards took place and this information is shown in Tables 3. The three most occurring countries on the PHE bulletin, USA (112 hazards), India (56) and China (34) all appeared in the top 10 countries of the rest of the databases. Moreover, the Philippines, Indonesia and Pakistan also appeared in the top 10 of all databases excluding NASA’s. However, both the United Kingdom (21) and Canada (20) appeared frequently in the PHE bulletin but were barely recorded in the other three databases with hazards in the United Kingdom being covered on four occasions and Canada three. Similarly, countries including Japan, Russia and Argentina were included in the top 10 countries from the other databases but were ranked much lower in the PHE Bulletin - 21st, 35th, and 40th respectively.

**Table 3:**
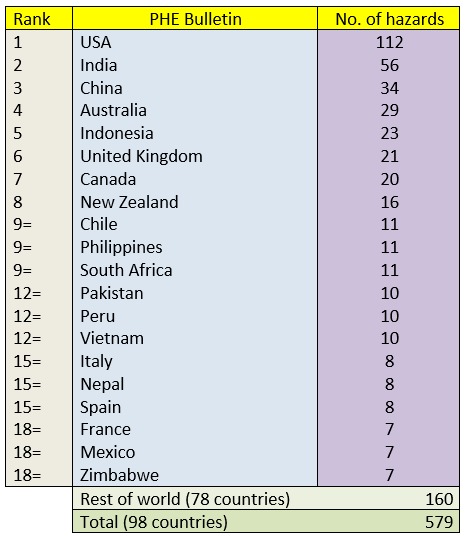
Number of unique hazards ranked by country of hazard in the PHE bulletin.

**Table 4:**
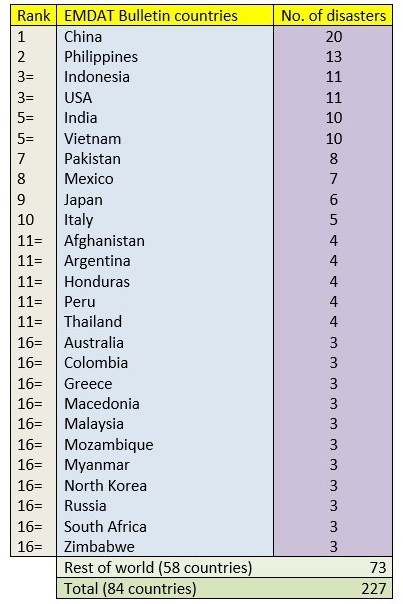
Number of unique hazards ranked by country of hazard in the EM-DAT database.

**Table 5:**
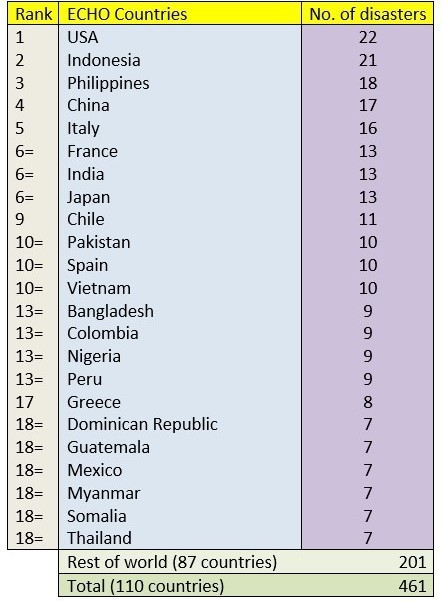
Number of unique hazards ranked by country of hazard in the ECHO database.

**Table 6:**
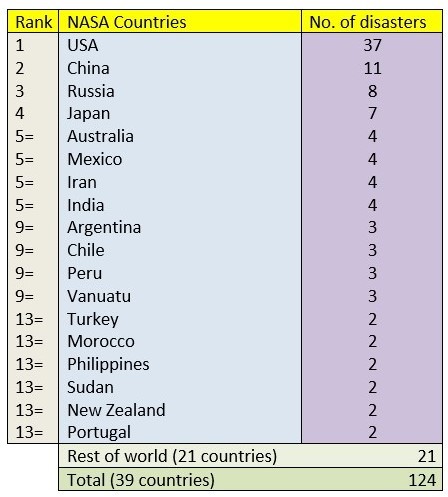
Number of unique hazards ranked by country of hazard in the NASA database.

## Discussion

**Summary of evidence**

Based on our findings, the PHE bulletin provides a thorough overview of hazards taking place in the world every week and it is comparable to global hazard or disaster databases such as those produced by EM-DAT, ECHO and NASA. It was found that the PHE bulletin and comparison databases appeared to over report hazards either occurring in developed countries or those considered fast-onset.

**Are some hazards favoured within the Bulletin?**

It is generally observed that across media outlets, there is very little correlation between the number of deaths attributable to a hazard and number of stories published on it 4,^13^. Indeed, using data from the PHE bulletin, we can see that the most commonly reported six hazard types - floods, convective storms, landslides, wildfires, cyclones and earthquake - are all rapidly evolving hazards. Moreover, in comparison, a slowly evolving hazard such as drought, which accounted for around 6% of confirmed global hazards from 2005-2015, only accounted for 4.6%, 0.4% and 2.8% of hazards reported in the PHE bulletin, EM-DAT and ECHO databases respectively. Therefore, one can theorise that the rapidly evolving events which receive increased coverage may do so at the expense of slow-onset hazards. This is despite these hazards often resulting in comparable consequences with respect to mortality and impact on a community 13,26,^27^.

CRED, which runs and populates the EM-DAT disaster database, describes a relative decrease in major disasters over the past 30 years; however, trends in individual disaster type vary, with the biggest increase in flooding and drought relative to geological disasters and windstorms 7. Fair disaster communication should reflect these trends and the proportionality of hazards around the world. In this sense, the PHE bulletin appears to reflect this. Table 7 illustrates that, compared to the World Disasters Report 2016, the proportion of each individual hazard type reported by PHE is similar. Continuing this theme, following a major hazard, the media tend to frame subsequent crises in relation to the previous hazard - for example, Hurricane Gustav following Hurricane Katrina. This can lead to public misinterpretation of the impact of an event and its aftermath can be ignored 28. Therefore, the idea of ‘newsworthy longevity’ and the disproportionate media coverage of rapidly evolving hazards should be important considerations when composing a truly comprehensive hazards bulletin.

**Table 7:**
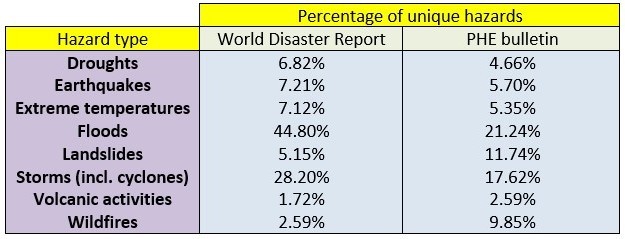
Comparison of the percentage of each hazard type in the World Disasters Reports 2016 and the PHE bulletin.

**Are some countries favoured within the Bulletin?**

The CRED Annual Disaster Statistics Review lists China, the USA and India as experiencing the greatest number of disasters in 2015 2. As depicted in Table 8, six of the top ten countries by number of reported hazards in the World Disaster Report 2015 were also in the bulletin’s ten most common countries for hazards. It is important to note that definitions of hazards can vary and, as the 2017 EM-DAT World Disaster Report is not published yet, data is not directly comparable. However, data has shown that, year on year, India, USA, China, Philippines and Indonesia repeatedly experience the highest number of hazards annually 2. Given that these five countries appear in the top ten countries included in the PHE bulletin, we can initially conclude that the bulletin content appears to be comparable to recognised global hazard reports.

**Table 8:**
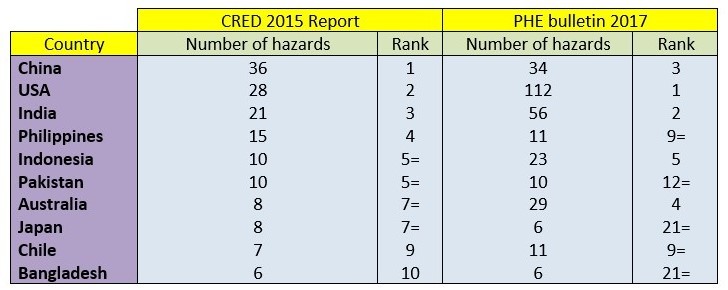
Comparison of the most common countries and number of hazards occurring in each country in the CRED 2015 Report and the PHE bulletin.

On the other hand, Table 9 which compares the location of hazards in the 2016 World Disaster Report to those in the PHE bulletin, suggests that the PHE bulletin under-reports those hazards occurring in Africa but over-reports those taking place in the Americas or Oceania. One hypothesis for this discrepancy may be that developed and largely English speaking countries such as Australia and New Zealand in Oceania and USA and Canada in the Americas are over-reported in the news and that this is then portrayed in the PHE bulletin content.

**Table 9:**
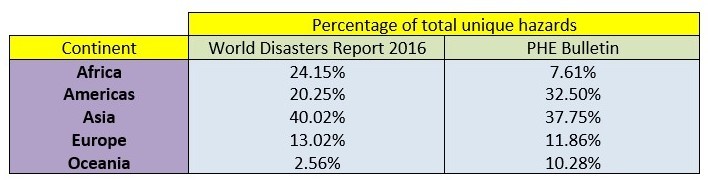
Comparison of the percentage of total hazards in each continent per the World Disasters Reports 2016 and the PHE bulletin.

The over or under-reporting of hazards has clear consequences. Evidence shows that the media hold significant leverage over how much support a country may receive in the wake of a hazard 8,11,^20^. For example, in the months after the December 2004 tsunami, for each news article published the ratio of donations increased by 3.15 relative to those on April 4th 2005 29. Therefore, clear communication of all hazards is imperative; even more so given that hazards often have a higher human impact in developing countries than in developed ones 9,26,^30^. Both the PHE bulletin and other databases should attempt to broadcast hazards irrespective of the location or developmental index of the country in which they are taking place.

**Limitations**

Technological hazards were not included in our comparison as the majority do not meet the PHE bulletin inclusion (due to the civil service code of conduct of remaining politically neutral) criteria of articles maintaining political neutrality 25.

The Google Alerts for compiling the PHE bulletin are only available from Monday to Friday; therefore, hazards that occur on weekends and that do not receive press coverage on the following Monday may not be included despite fulfilling all inclusion criteria. Moreover, the PHE bulletin is reliant purely on Google Alerts to acquire sources and so some hazards may be missed if they occur in areas where Google has less prominence or where there is less media coverage.

Lastly, non-English language articles were excluded as translation facilities were not available. This may explain in part why there appeared to be a greater proportion of hazards covered in Canada and the United Kingdom and fewer hazards in Russia and Japan compared to EM-DAT, ECHO and NASA databases.

**Recommendations**

The following are the recommendations resulting from our research. We recommend that the resources compared (the PHE bulletin, EM-DAT, ECHO and NASA) review their inclusion criteria and assess whether the discrepancies in hazard type and country discussed earlier can be ratified through changes in how hazards are assessed for inclusion. We would also encourage media outlets, where possible, to increase coverage of slowly evolving hazards, especially those in developing countries where increased coverage can have immense impacts on the long-term consequences of hazards.

## Competing Interests

The authors declare that there are no competing interests.

## Data Availability Statement

All relevant data are within the manuscript file.

## Corresponding Author

Thomas Richardson is the Corresponding Author and can be reached via thomas.richardson9@nhs.net or thomas.richardson4@outlook.com.

## Appendix 1:

**Google Alerts for PHE Bulletin**

“drought” “water”

“extreme cold”

“cyclone” “weather”

“flood” “rain”

“forest fire”

“hailstorm”

“heatwave” “heat wave”

“high winds”

“Hurricane”

“landslide” “weather”

“lightning” “damage”

“monsoon rain”

“sand storm”

“Sendai framework”

“smog”

“storm” “wind” “rain”

“strong winds”

“thunderstorm”

“tornado” “wind”

“torrential rain”

“Typhoon” “storm” “wind”

“volcanic ash”

“waterspout”

“bushfire”

Disaster “Science and Technology”

“heavy rain landslide flood”

UNISDR “Science and Technology”

“volcano eruption”

"wildfires"

## Appendix 2:

**Guidance on the PHE Global Hazards Bulletin**

The bulletin is sent once a week, usually on a Friday morning. The bulletin is populated with information collected for a variety of sources but with its main source of information being Google Alerts set up on the UNISDR.Stag Gmail account (log in details available on request).

This document is intended to provide high level guidance on writing and distributing the bulletin. A template version of the bulletin is attached as Annex 1.

**What to include:**

-Events occurring within the previous 7 days (can be longer if slow-onset)

-Natural hazards (earthquake, hurricane, typhoon, sand storm, blizzard, drought etc.)

-Technological hazards (Industrial incidents, infrastructure failures etc.)

-Public health emergencies (Ebola outbreak)

**What not to include:**

-Anything overtly political (war, ‘terror’ attacks, riots or civil disobedience)

-Events with limited human impact (events in non-populated areas)

-Stories overly critical of a Member States response to the event.

**What to seek further advice on:**

-States with debated legality (Palestine for example)

-Stories concerning UK incidents during periods of sensitivity around National or Local elections.

**Thresholds**

-For inclusion in the bulletin, the event needs to have a direct or indirect impact on a number of people. This number will be different depending on whether that impact is direct or indirect:

-Direct impacts – **greater than three impacted** - Fatalities caused directly by the event; Injuries caused by the event

-Indirect impacts - ****greater than 100 impacted - ****Infrastructure failure (road closed, power outage); Curfew or quarantine

**Sources**

Include:

-Broadsheets (both UK titles and overseas. If unsure, check title on Wikipedia)

-Official press releases from government (given the information is partial and not overly political (party politics). Can be either national or local)

-Information from international agencies (UN family, INGOs)

-Scientific journals

Avoid:

-Tabloid journalism (Both UK and overseas titles. If unsure, check title on Wikipedia)

-Blogs (unless data is verified with links to respected sources)

-Social media (unless data is verified with links to respective sources)

## 
